# Preliminary outcomes from a single-session, asynchronous, online stress and anxiety management workshop for college students

**DOI:** 10.47626/2237-6089-2021-0448

**Published:** 2023-09-22

**Authors:** Carol S. Lee, Margaret Bowman, Jenny L. Wu

**Affiliations:** 1 Department of Psychology Nevada State College Henderson NV USA Department of Psychology, Nevada State College, Henderson, NV, USA.; 2 Department of Clinical Psychology University of Massachusetts Boston Boston MA USA Department of Clinical Psychology, University of Massachusetts Boston, Boston, MA, USA.

**Keywords:** Mental Health, stress, anxiety, dialectical behavior therapy, online intervention

## Abstract

**Objectives:**

Self-guided, asynchronous, online interventions may provide college students access to evidence-based care, while mitigating barriers like limited hours of service. Thus, we examined the preliminary effectiveness of a 45-minute, self-guided, asynchronous, online, dialectical behavior therapy (DBT)-informed stress and anxiety management workshop. College undergraduates (n = 131) were randomized to either workshop (n = 65) or waitlist control (n = 66) conditions.

**Methods:**

Participants in the workshop condition completed baseline measures of depression, stress, and anxiety, before completing the workshop. Participants in the waitlist control condition only completed the baseline measures. All participants were reassessed at 1-week follow-up.

**Results:**

Controlling for baseline measures, students in the workshop condition experienced significantly less stress and greater self-efficacy to regulate stress and anxiety at follow-up, compared to waitlist controls.

**Conclusion:**

A 45-minute, self-guided, asynchronous, online DBT skills-informed stress and anxiety management workshop may reduce stress and improve self-efficacy to regulate stress and anxiety.

## Introduction

Research consistently indicates the presence of a mental health crisis among college students in the United States, with recent estimates of over 75% of US college students experiencing clinically significant stress and anxiety.^1^ Such findings are likely the result of the multiple stressors that college students, and particularly diverse and non-traditional college students, face including identity development, academic and familial demands, financial strain, and discrimination.^2-4^ Notably, experiences of stress and anxiety have been positively associated with a wide range of academic and mental health difficulties, including anxiety disorders, depressive disorders, eating disorders, substance abuse, and suicidality.^5,6^ Despite this, a high percentage of US college students do not seek mental health services, with the majority of students citing mental health stigma, busy schedules, and limited hours of services as the primary barriers to doing so.^7-9^

To address these barriers and to improve the accessibility of evidence-based mental health support, there has been an increase in development and implementation of self-guided asynchronous online stress and anxiety management interventions for college students.^10-18^ Here, an asynchronous intervention refers to the lack of any real-time or in-person facilitation, support, or guidance from a facilitator or researcher. Additionally, self-guided refers to the lack of any facilitator or coach included with the intervention. As such, self-guided, asynchronous, online interventions can both increase accessibility and decrease barriers to mental health support, as they can be completed at an individual’s own pace, at any time, and at any location with internet access. Strikingly, studies thus far indicate that such interventions result in a variety of positive mental health outcomes, including lower anxiety,^11,12,14,17^ lower stress,^10,14^ lower depression,^11,14,17,18^ greater psychological wellbeing,^15^ and greater psychological flexibility.^16^

The positive outcomes indicate that self-guided, asynchronous, online stress and anxiety management interventions offer a way for students to access effective services, while mitigating barriers like limited hours of service, transportation, travel time, and the mental health stigma of physically seeking services.^19^ However, most interventions have limited accessibility by requiring participants to attend multiple sessions, thus placing greater time and scheduling burden on participants. Although one intervention was single-session,^11^ the researchers required participants to complete the intervention in the lab, thus reducing external validity and excluding students experiencing the time, transportation, and scheduling barriers noted above. As such, there has not yet been an examination of a single-session, self-guided, asynchronous, online stress and anxiety management intervention delivered outside of the lab that further addresses the common barriers that many college students face.

One method of achieving this may be to implement a workshop-style intervention based on acceptance-based approaches and strategies, such as acceptance-based behavioral therapy (ABBT),^20^ acceptance and commitment therapy (ACT),^21^ or dialectical behavior therapy (DBT),^22,23^ which have demonstrated promising therapeutic outcomes. Although acceptance-based workshop approaches have yet to be studied in a self-guided, asynchronous, online format, they have been shown to have positive outcomes when delivered in-person to college students.

For example, Brown et al.^24^ utilized a 2-hour ACT-based test anxiety workshop and found a significant increase in test performance and trend toward decreased test anxiety.^24^

Similarly, other studies indicate that, following an ABBT-based stress and anxiety management workshop, symptoms of stress and anxiety decreased at 1-month follow-up,^25^ and depression severity decreased at 3-month follow up.^26^ There is also emerging evidence for the benefits of DBT as a single-session intervention,^27^ as well as evidence for promising outcomes of abbreviated DBT-informed skills groups for college students, such as greater perceived emotion regulation and reductions in anxiety, stress, and depression.^28-31^

In sum, despite a high prevalence of stress and anxiety, many US college students do not seek mental health services due to mental health stigma, busy schedules, and being unavailable during hours of services.^7-9^ Self-guided, asynchronous, online interventions and single-session workshops have been proposed as a means of providing mental health intervention while overcoming some of these practical barriers.^10-18,24-26^ The present study thus utilized a randomized waitlist control design to examine the preliminary effectiveness of a 45-minute, DBT-informed, stress and anxiety management workshop delivered asynchronously online. We hypothesized that, controlling for baseline measures, students in the workshop group would experience lower depression, anxiety, and stress compared to those in the waitlist control group at 1-week follow-up. Additionally, we hypothesized that, controlling for baseline measures, students in the workshop group would experience greater self-efficacy to regulate stress and anxiety compared to the waitlist control group at 1-week follow-up.

## Methods

### Participants

**Figure 1:**
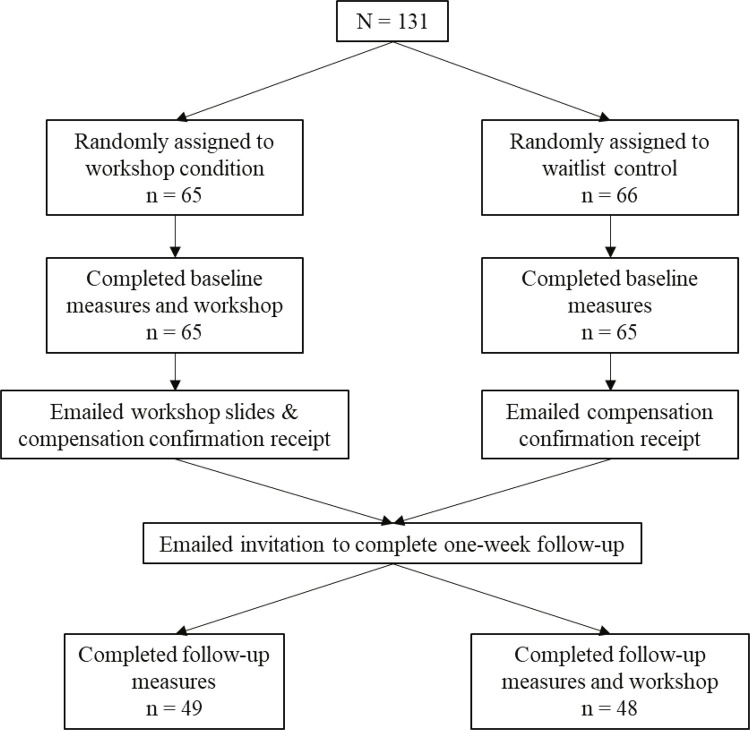
Flow chart representing study methodology and participant flow through conditions.

The study protocol was approved by the institutional review board of the Nevada State College, Henderson, NV, USA. We advertised the study on an open-access 4-year college campus using e-mails and course announcements advertising a two-part online Qualtrics study about a “stress and anxiety management workshop”. The study was open to all individuals meeting the inclusion criteria of being a current student aged from 18 to 65. All participants were randomly assigned by Qualtrics to the workshop or waitlist control conditions. A total of 131 participants (workshop = 65; waitlist control = 66) completed part one of the study, 97 of whom (workshop = 49; waitlist control = 48) completed part two at 1-week follow-up. The participant flowchart is shown in Figure 1. Study completers and non-completers were compared at baseline and no significant differences were found in any outcome measures (p values = 0.24-0.85). The demographic characteristics are shown in Table 1.

**Table 1:** Demographic characteristics

Demographic	Workshop n (%)	Control n (%)	p-value	Total sample n (%)
Gender identity			0.11	
Male	7 (10.77)	15 (22.73)		22 (16.79)
Female	55 (84.62)	51 (77.27)		106 (80.92)
Transgender	1 (1.54)	0 (0)		1 (0.76)
Other*	2 (3.08)	0 (0)		2 (1.53)
Sexual orientation			0.50	
Heterosexual	49 (75.38)	55 (83.33)		104 (79.39)
Gay/lesbian	4 (6.15)	2 (3.03)		6 (4.58)
Bisexual	7 (10.77)	6 (9.09)		13 (9.92)
Other^†^	5 (7.69)	2 (3.03)		7 (5.34)
Race^‡^				
Alaskan native/native American/Indigenous^§^	2 (3.08)	0 (0.00)	0.15	2 (1.53)
Asian	3 (4.62)	8 (12.12)	0.12	11 (8.40)
Black	10 (15.38)	10 (15.15)	0.97	20 (15.27)
Latino (not White)	20 (30.77)	18 (27.27)	0.66	38 (29.01)
Latino (White)	13 (20.00)	15 (22.73)	0.70	28 (21.37)
Pacific Islander	1 (1.54)	3 (4.55)	0.32	4 (3.05)
White	15 (23.08)	21 931.82)	0.18	35 (26.72)
Multiracial	6 (9.23)	5 (7.58)	0.73	11 (8.40)
Therapy experience			0.23	
No, never	32 (49.23)	39 (59.09)		71 (54.20)
Yes, in the past	22 (33.85)	21 (31.82)		43 (32.82)
Yes, currently	11 (16.92)	5 (7.58)		16 (12.21)

N = 131 (workshop = 65, control = 66).

p-values reflect the statistical comparisons between groups.

* Identities endorsed: gender fluid, prefer not to respond

^†^ Identities endorsed: fluid, grey asexual, pansexual, queer, prefer not to respond

^‡^ Participants could endorse multiple identities

^§^ Tribal affiliations endorsed: Cherokee

### Measures

#### Depression, Anxiety, and Stress Scales – 21 item (DASS-21)^32^

This 21-item self-report measure consists of three subscales: the depression subscale (DASS-D), the anxiety subscale (DASS-A), and the stress subscale (DASS-S). Each subscale is examined to measure symptoms of depression (DASS-D), situation-specific affective symptoms of anxiety (DASS-A), and generalized physiological symptoms of anxiety (DASS-S). Participants rated items on a scale from 0 (did not apply to me at all) to 3 (applied to me very much or most of the time). We used the DASS-21 to measure participants’ symptoms of depression, anxiety, and stress at baseline and at 1-week follow-up. The DASS-21 has been shown to have good convergent and discriminant validity and good internal consistency.^33^ Similarly, the measure demonstrated good internal consistency in our sample at baseline (α = 0.94) and at 1-week follow-up (α = 0.95).

#### Anxiety Self-Efficacy (ASE)

The ASE is a two-item, self-report measure created for this study, assessing respondents’ perceived ability to manage and tolerate anxiety. Participants utilized a 1 (not at all) to 9 (very) Likert response scale. All items were taken from the Self-Efficacy for Social Situation Scale (SESS)^34^ and modified to be about stress and anxiety tolerance and management skills (e.g.: “Is it possible for you to tolerate and manage stress and anxiety well, despite any weaknesses you might have in stress and anxiety tolerance and management skills?”) rather than about social skills (e.g.: “Is it possible for you to perform well in social situations, despite any weaknesses you might have in social skills?”). The ASE was administered to assess participants’ self-efficacy to tolerate and manage stress and anxiety at baseline and at 1-week follow-up. The ASE demonstrated good internal consistency in our sample at baseline (α = 0.82) and at 1-week follow-up (α = 0.87)

## Stress and Anxiety Management Workshop

The self-guided Stress and Anxiety Management Workshop was delivered asynchronously through Qualtrics and consisted of eight videos recorded by the first and second authors. To maximize student attention to the workshop and to reduce fatigue, all videos were less than 5 minutes long (range: 1 minute and 10 seconds to 4 minutes and 49 seconds). Each video was also followed by one to three engagement or reflection questions, which asked participants to reflect on their experiences with stress and anxiety and how they could apply each skill learned over the next week and to their daily lives. Each video and its corresponding engagement questions were presented on their own page. To ensure that participants watched the videos, each page was timed to the length of the video so that participants could not move on until the allotted time had passed. Participants were able to re-watch videos.

The workshop content was adapted from the distress tolerance and emotion regulation modules of the DBT skills manual.^22,23^ Participants were first provided psychoeducation on the prevalence, consequences, symptoms, and function of stress and anxiety. Participants then learned about methods of tolerating their stress and anxiety in the moment by learning and practicing the TIPP skills.^23^ Participants also learned about methods of reducing vulnerability to stress and anxiety by learning the ABC Please skill.^23^ We chose to focus the workshop on teaching participants skills for tolerating and regulating stress and anxiety, given research on their negative associations with stress and anxiety.^35-37^

## Procedures

After providing informed consent, all participants were randomly assigned to either the workshop or waitlist control condition by Qualtrics. Participants in the workshop condition completed the baseline measures of the demographics questionnaire, the DASS-21, and the ASE, then completed the asynchronously delivered self-guided stress and anxiety management workshop. Participants in the waitlist control condition only completed the baseline measures.

Participants in both groups were then informed that they would receive a link to part two of the study in 1 week and be compensated with course research credit or a $10 Tango Rewards gift card, which allows participants to receive a gift card for a vendor of their choice. All participants were then automatically emailed a receipt for the compensation. Participants in the workshop condition also received a copy of the workshop slides.

After 1 week, participants were emailed a link to part two, and were asked to complete part two within the next 3 days, as the link to part two expired after 3 days. We chose to set an expiration date on the link to control for time elapsed since part one. Participants on the workshop condition completed DASS-21 and ASE follow-up measures and participants on the waitlist control condition completed the follow-up measures and the workshop. Participants were again compensated with either course research credit or a $10 Tango Rewards gift card and automatically emailed a compensation receipt. Participants in the waitlist control condition also received a copy of the workshop slides.

## Statistical analysis

Our descriptive statistics were reported as frequencies, percentages, and means and standard deviations. To examine differences between outcome and demographic variables at baseline, we conducted *t* tests for continuous variables and chi-square analyses for categorical variables. For our primary analysis, we conducted a multivariate analysis of variance (MANCOVA) to examine differences in outcome variables at 1-week follow-up between conditions, with baseline scores of all outcome variables entered as covariates. Finally, to examine relations between outcome variables, we calculated residualized gain scores and conducted a correlation analysis. All data were analyzed using the Statistical Package for the Social Sciences (SPSS) version 22 and the significance level adopted was 0.05.

## Results

### Preliminary analyses

Table 2 provides the descriptive statistics for all outcome variables. All skewness and kurtosis values were within acceptable ranges. To examine differences between outcome and demographic variables at baseline, we conducted *t* tests for continuous variables and chi-square analyses for categorical variables. Although participants were randomized to the groups, initial pre-workshop scores on the DASS-D and DASS-S were significantly higher in the workshop group than in the waitlist control group (t[129] = -2.46, p = 0.02; t[129] p = 0.02, respectively). Scores on the DASS-A and the ASE did not differ significantly between groups (p = 0.17; p = 0.36, respectively). Baselines scores between groups also did not differ between age, gender, sexual orientation, race, or therapy experience (p values = 0.11-0.97).

**Table 2:** Outcome variables by condition

	Pre M (SD)			1 Week Follow-up M (SD)				
				
Measure	Workshop	Control	Total	Workshop	Control	Total	F(1, 91)	ηp^2^
DASS (Depression)	6.55 (5.47) n = 65	4.36 (4.69) n = 66	5.45 (5.19) n = 131	4.76 (4.63) n = 49	3.69 (4.26) n = 48	4.23 (4.46) n = 97	2.87	0.03
DASS (Anxiety)	6.66 (5.25) n = 65	5.45 (4.64) n = 66	6.05 (4.97) n = 131	4.80 (5.00) n = 49	4.65 (4.44) n = 48	4.72 (4.71) n = 97	3.20	0.03
DASS (Stress)	8.57 (5.10) n = 65	6.55 (4.78) n = 66	7.55 (5.02) n = 131	6.35 (4.40) n = 49	6.17 (4.59) n = 48	6.26 (4.48) n = 97	13.42*	0.13
ASE	11.29 (3.32) n = 65	11.74 (3.37) n = 66	11.52 (3.34) n = 131	13.78 (2.58) n = 49	12.71 (3.35) n = 48	13.25 (3.02) n = 97	13.60*	0.13

Note: DASS = Depression Anxiety and Stress Scale, ASE = Anxiety Self-Efficacy

* p < 0.001

### Primary analyses

We conducted a MANCOVA to test the hypothesis that, compared to waitlist control, students in the workshop group would experience greater decreases in depression, anxiety, and stress, and greater increases in self-efficacy to regulate stress and anxiety. The independent variable was condition (workshop group versus waitlist control group), and the dependent variables were DASS-D, DASS-A, DASS-S, and ASE scores at 1-week follow-up (Table 2). To adjust for baseline scores, baseline scores of all four outcome measures were entered as covariates. The results indicated that, controlling for baseline measures, the combined dependent variables were significantly different between groups with a large effect size, F(4, 88) = 4.98, p = 0.001, ηp^2^ = 0.18. Univariate analyses indicated that, controlling for baseline measures, students in the workshop group experienced significantly lower DASS-S scores with a medium effect size, F(1, 91) = 13.42, p < 0.001, ηp^2^ = 0.13, and significantly higher ASE scores with a medium effect size, F(1, 91) = 13.60, p < 0.001, ηp^2^ = 0.13. In contrast, there was no significant difference between groups in DASS-D scores, F(1, 91) = 2.87, p = 0.09, ηp^2^ = 0.03, or DASS-A scores, F(1, 91) = 3.20, p = 0.08, ηp^2^ = 0.03.

### Additional analyses

Finally, given the findings indicating that only the DASS-S and ASE were significantly different between groups, as well as the literature implicating self-efficacy as an important mechanism of change in therapeutic outcomes, we examined the relations between the residualized gain scores, which adjust for baseline scores, for the DASS-D, DASS-A, DASS-S, and the ASE.

The findings indicated that the ASE residualized gains score was significantly associated with the DASS-S, r = -0.36, p < 0.001, but not the DASS-D, r = -0.16, p = 0.12, or the DASS-A, r = -0.20, p = 0.052.

## Discussion

To increase accessibility to evidence-based interventions for stress and anxiety in college students, researchers have examined the positive outcomes of self-guided interventions delivered asynchronously online, single-session acceptance-based workshops, or abbreviated DBT skills-informed interventions. As such, the purpose of this study was to examine the preliminary effectiveness of a self-guided, DBT skills-informed stress and anxiety management workshop delivered asynchronously online. We hypothesized that, compared to waitlist controls, students completing the workshop would experience greater self-efficacy to regulate stress and anxiety, as well as lower depression, anxiety, and stress at 1-week follow-up. The results indicated that, controlling for baseline scores, participants in the workshop condition experienced significantly less stress and significantly greater self-efficacy to regulate stress and anxiety at 1-week follow-up compared to waitlist controls. This is consistent with the literature, which has similarly indicated that stress decreased^25,30^ and perceived emotion regulation increased^28-31^ following completion of an abbreviated acceptance-based intervention.

Of note, both the previous literature and the present study utilized the DASS-S to measure stress, which has been most closely related to physiological symptoms of generalized anxiety.^32,38^ As such, results may indicate that the workshop was effective in increasing participants’ perceived ability to manage stress and anxiety, and in reducing physiological symptoms of generalized anxiety at 1-week follow-up. This is consistent with the workshop content, which focused on methods of reducing physiological arousal in the moment (via the TIPP skill) and on incorporating behavioral changes to promote physical and mental health and resiliency (via ABC Please).

Contrary to our hypothesis, the results also indicated that depression and anxiety were not significantly lower at 1-week follow-up compared to waitlist controls. Of note, the present study utilized the DASS-A to measure anxiety, which has been most closely related to situation-specific experiences of anxiety and fear (e.g., panic, social anxiety, phobias).^32^ As such, it is possible that our workshop, while targeting physiological symptoms of generalized anxiety, does not target situation-based fear and anxiety due to its delivery, length, and/or content. For example, unlike previous interventions that resulted in lower depression^26^ or lower anxiety^25,29,31^ our workshop was conducted asynchronously online. Thus, it is possible that our workshop allowed for social isolation and decreased behavioral activation in a way that in-person interventions, which provide social support and an opportunity for behavioral activation, do not.

Our workshop was also significantly shorter (45 minutes) than previous interventions (range: 2-18 hours) and did not include mindfulness or interpersonal effectiveness skills, which may better target situation-based fear and anxiety.

Finally, our additional analyses indicated self-efficacy to regulate stress and anxiety was significantly associated with our measure of stress, but not depression or anxiety. Given self-efficacy’s role as an important mechanism of psychotherapeutic change,^39-42^ these findings further indicate that our workshop was successful in targeting physiological symptoms of generalized anxiety, but not depression or situation-based fear and anxiety. To examine changes in all three constructs, while maximizing accessibility, future research should explore use of a more comprehensive intervention that still adheres to a brief, self-guided and asynchronous format.

### Limitations and future directions

Our study should be considered in the context of several limitations. Importantly, we utilized a wait-list control design, in which participants in the control group did not receive a placebo or alternate intervention. As such, it is possible that the results are a due to engagement with a workshop, rather than the workshop content. Further research should thus utilize a placebo or a workshop comparison group. Although we utilized Qualtrics’ timed questions to control for engagement, we also ultimately could not fully control for participants’ engagement with the material or the presence of environmental distractions. Although this format ultimately increased external validity, we recognize that engagement with material likely impacts the extent to which participants experienced gains. Our workshop also utilized an adapted measure for self-efficacy, which was not evaluated for reliability and validity, outside of internal consistencies. However, the measure has been historically rated by a team of research assistants as having good face validity and was adapted from an established measure. Additionally, our study had relatively high attrition from part one to 1-week follow-up (25.95%). Although study non-completers did not significantly differ from study completers by condition or baseline measures, we cannot make conclusions about reasons for drop-out and future research should examine reasons for drop-out.

Several factors also impacted the generalizability of our findings. For example, because we only conducted a 1-week follow-up, we were unable to assess any long-term or sustained impacts of our workshop. As such, findings cannot be generalized as long-term changes and future research should examine outcomes after several months or years to do so. Finally, because students were recruited from a small, open-access college and were not a clinical sample, the findings cannot be generalized to other institutions or clinical samples.

### Clinical implications

Despite its limitations, our findings ultimately suggest that utilizing a 45-minute self-guided DBT skills-informed workshop delivered asynchronously online can decrease physiological symptoms of generalized anxiety, but not symptoms of depression or situation-specific fear and anxiety. While further research and development is thus necessary to maximize outcomes and generalizability, the findings provide an encouraging outlook on the potential use of this modality as an easily distributed, low cost, and accessible way to disseminate evidence-based skills to student populations.
